# Achievements and Future Perspectives of the Trivalent Thulium-Ion-Doped Mixed-Sesquioxide Ceramics for Laser Applications

**DOI:** 10.3390/ma15062084

**Published:** 2022-03-11

**Authors:** Angela Pirri, Roman N. Maksimov, Jiang Li, Matteo Vannini, Guido Toci

**Affiliations:** 1 Istituto di Fisica Applicata “N. Carrara”, Consiglio Nazionale delle Ricerche, 50019 Sesto Fiorentino, FI, Italy; 2 Institute of Electrophysics UrB RAS, 620016 Ekaterinburg, Russia; romanmaksimov@e1.ru; 3 Ural Federal University Named after the First President of Russia B.N. Yeltsin, 620002 Ekaterinburg, Russia; 4 Key Laboratory of Transparent Opto-Functional Inorganic Materials, Shanghai Institute of Ceramics, Chinese Academy of Sciences, Shanghai 201899, China; lijiang@mail.sic.ac.cn; 5 Center of Materials Science and Optoelectronics Engineering, University of Chinese Academy of Sciences, Beijing 100049, China; 6 Istituto Nazionale di Ottica, Consiglio Nazionale delle Ricerche, INO-CNR, 50019 Sesto Fiorentino, FI, Italy; matteo.vannini@ino.cnr.it (M.V.); guido.toci@ino.cnr.it (G.T.)

**Keywords:** solid-state lasers, rare-earth doped laser materials, infrared laser, diode-pumped lasers, thulium lasers, thulium-doped mixed laser ceramics, mixed-sesquioxide ceramics

## Abstract

This paper is devoted to reviewing the latest results achieved in solid-state lasers based on thulium-doped mixed-sesquioxide ceramics, i.e., (Lu,Sc,Y)_2_O_3_. The near- and mid-infrared regions are of interest for many applications, from medicine to remote sensing, as they match molecular fingerprints and cover several atmospheric transparency windows. These matrices are characterized by a strong electron–phonon interaction—which results in a large splitting of the ground state—and by a spectral broadening of the optical transition suitable for developing tunable and short-pulse lasers. In particular, the manuscript reports on the trivalent thulium laser transitions at 1.5, 1.9, and 2.3 µm, along with the thermal and optical characteristics of the (Lu,Sc,Y)_2_O_3_ ceramics, including the fabrication techniques, spectroscopic and optical properties, and laser performances achieved in different pumping regimes, such as continuous-wave (CW), quasi-CW, and pulsed modes. A comparison of the performance obtained with these mixed-sesquioxide ceramics and with the corresponding crystals is reported.

## 1. Introduction

Over the last several years, a large number of scientific papers focused on trivalent thulium ions (Tm^3+^) as activators in laser-grade materials have been published, because Tm^3+^, together with Ho^3+^ [1], is an excellent candidate for the development of laser sources emitting in the near- and mid-infrared, from 1.5 to 2.3 µm [2]. As a matter of fact, the most common bulk matrices (i.e., crystals and transparent polycrystalline ceramics [3])—such as YAG [3,4,5,6,7,8,9,10,11], Lu_2_O_3_ [12,13,14,15,16], Sc_2_O_3_ [17,18,19,20,21], Y_2_O_3_ [22,23,24,25], YLF [26,27], CaF_2_ [28,29,30], LuAG [31,32,33]—were developed and studied achieving good performance in terms of laser output power, short pulse duration, and broad range of tunability (from 1.84 to 2.07 µm).

What makes trivalent thulium ions even more interesting is the possibility to excite the Tm^3+^ laser transitions at 2 µm [3] and 2.3 µm [34] by means of high-efficiency, commercially available semiconductor-based pump sources, exploiting the strong absorption band located at 790–800 nm [35]. Moreover, as the corresponding full width at half-maximum (FWHM) is in the range of a few tens of nanometers (i.e., 25–40 nm), tight control of the wavelength peak of laser diodes (LDs) is not mandatory. In the past, complex and more expensive pumping laser systems such as Ti:sapphire were used.

As many applications require laser emission at longer wavelengths—as will be clarified later on—some crystals [36,37,38,39,40,41,42] and mixed ceramics have attracted the interest of the scientific community because they are characterized by slightly broader and smoother gain spectra, which can be exploited in developing tunable and sub-100 fs laser systems [43,44,45].

Among outstanding hosts, many research groups have focused their attention on three cubic sesquioxide ceramics [46]—i.e., Lu_2_O_3_ [14,15,16], Sc_2_O_3_ [21], and Y_2_O_3_ [22]—because they can form solid solutions—i.e., (Lu,Sc,Y)_2_O_3_—maintaining excellent thermomechanical properties and optical quality. It is well known that sesquioxide ceramics show high thermal conductivity, a high refractive index (i.e., 1.94 at 800 nm and 1.92 at 2066 nm in 2*at.*% Tm:Lu_2_O_3_ [16]), a broad transparency range (0.22–8 µm), a positive *dn*/*dt* thermo optical coefficient (i.e., (2.0 ± 0.5) × 10^−5^ K^−1^ in 2*at.*% Tm:Lu_2_O_3_ [47]), low-energy phonons, and a high nonlinear refractive index (at 2070 nm n_2_ = 3.3 × 10^−16^ cm^2^/W [15] and 8.6 × 10^−16^ cm^2^/W at 1064 nm [48]). Moreover, they support high concentrations of dopants, preserving their optical quality and good values of thermal conductivity in comparison with the undoped compositions. In particular, the thermal conductivity of Lu^3+^-based matrices is almost insensitive to the concentration of dopants, as Tm^3+^ and Lu^3+^ have similar ionic radii (i.e., r_ion_(Tm^3+^) = 0.0869 nm and r_ion_(Lu^3+^) = 0.0861 nm for VI-fold oxygen coordination [49]) and mass [50]. Furthermore, sesquioxides are characterized by a strong Stark splitting of manifolds, which results in an emission wavelength longer than 2050 nm. They have been tested in CW, Q-switching (100 ns at 2066 nm in Tm:Lu_2_O_3_ [16]), and mode-locking regimes (180 fs at 2070 nm 2*at.*% Tm:Lu_2_O_3_ [15]). Tm^3+^-doped sesquioxides are still a lively field of investigation.

Thanks to many efforts made to improve the manufacturing techniques, in recent years Tm^3+^-doped mixed-sesquioxide ceramics such as (Lu,Y)_2_O_3_ [51,52], (Sc,Y)_2_O_3_ [53], and (Lu,Sc)_2_O_3_ [54,55,56], as well as garnets (i.e., YSAG [57,58]), have been fabricated and their potentiality tested, finding excellent results. For instance, pulses as short as 54 fs were generated in Tm:(Lu_2/3_Sc_1/3_)_2_O_3_ [52], and a CW tuning range of 200 nm was measured in Tm:LuYO_3_ [59].

Regarding their applications, infrared laser sources are currently used in many technological applications [60], such as material processing [61], remote sensing [62,63], gas monitoring [60], medicine [64,65], and optical communication systems. All are triggered by two important properties of the infrared spectral range: first, many roto-vibrational absorption lines of various molecules are located in this range, known as the molecular fingerprint region; secondly, it contains several atmospheric transparency windows [66]. Moreover, 2 µm range radiation, known as the “*eye-safe region*”, plays a crucial role in all applications where eye safety is strategic; this is because the vitreous body of the human eye absorbs the radiation in this spectral range, preventing damage to the retina.

For example, radiation below 2.3 µm matches the absorption lines of methane and carbon dioxide [67], while in medicine it allows noninvasive measurements of glucose levels [68]; its strong absorption in water is attractive for surgical applications, since its depth of penetration into biological tissues is a few hundred micrometers, enabling microsurgical approaches (i.e., at 2.09 µm and 2.02 µm the penetration depth in water is 300 µm and 180 µm, respectively). Two-micrometer high-power laser systems can serve as pump sources for mid-infrared optical parametric oscillators (OPOs) [69] which, in turn, allow generation of laser wavelengths from 3 to 12 µm [70]. An interesting example is ZnGeP_2_-based OPOs, which are pumped at 2.1 µm and emit in the range from 3 to 5 µm [71]. Radiation at 1.5 μm is a useful wavelength for optical communication systems because it belongs to the S-band, i.e., 1.47–1.52 μm; it can be used as a laser pump for erbium-doped fiber amplifiers, as well as for Raman fiber amplifiers.

The main purpose of this paper is to review the important goals achieved with (Lu,Y)_2_O_3_, (Lu,Sc)_2_O_3_, and (Sc,Y)_2_O_3_ mixed-sesquioxide ceramics doped with Tm^3+^ ions. The full potential of these matrices is still to be explored. The knowledge of the state of the art can be useful to focus on future goals. This article is organized as follows: First, the main conditions for exciting a selected laser transition of Tm^3+^ are discussed. Secondly, for each mixed-sesquioxide ceramic, we report and discuss their spectroscopic and optical properties, as well as their laser behavior in CW and pulsed regimes. The results are then compared with those obtained in pure crystals or ceramics (whenever possible), because this paves the way for understanding how the induced disorder in lattices, by substituting some ions of the ordered host, influences the laser behavior of the mixed matrices.

## 2. Tm^3+^ Ion Laser Transitions: General Considerations

Thulium is the least abundant chemical element among those belonging to the rare-earth family (REEs); it is never found in pure form, and in 1911 it took Charles James around 15,000 recrystallizations of Tm(BrO_3_)_3_ to obtain a spectroscopically pure sample [72]. Thulium has many isotopes, of which only one is stable: natural Thulium is composed entirely by ^169^Tm.

**Figure 1:**
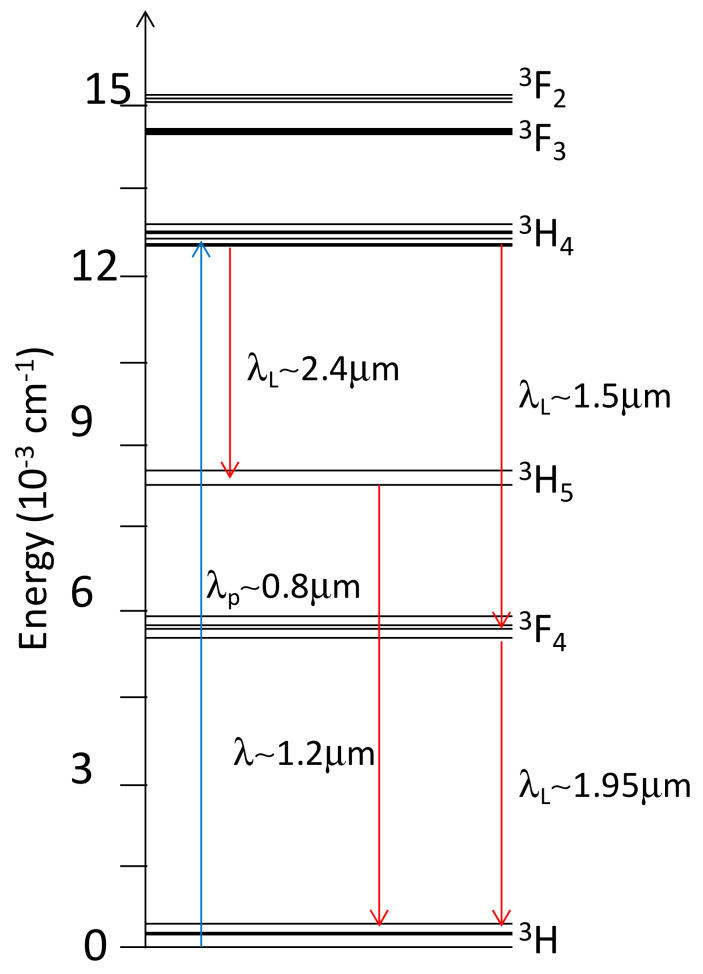
Energy level scheme of Tm^3+^ in Lu_2_O_3_ (adapted from [47]).

The trivalent ion, Tm^3+^, with an electron configuration [Xe]4f^12^, is employed in solid-state laser physics because of its three active optical transitions in near- and mid-infrared—i.e., ^3^H_4_ → ^3^H_5_, ^3^F_4_ → ^3^H_6_, and ^3^H_4_ → ^3^F_4_—with emissions centered at around 2.3 µm, 1.9 µm, and 1.5 µm, respectively. As the energy diagram of Tm^3+^ is extremely complex [73,74], a specific oscillation wavelength can be reached only if some conditions are fulfilled, since Tm^3+^ metastable states are involved in energy transfer processes such as non-radiative decay, excited-state absorption (ESA), and upconversion processes that take place. In Figure 1, the energy level scheme of Tm^3+^-doped Lu_2_O_3_ is reported.

Secondly, the Stark splitting of the Tm^3+^ manifolds is tightly connected to the host crystal field. In the most commonly used garnets, such as YAG or LuAG, the value of the splitting is lower than in mixed sesquioxides; for this reason, with the garnets, the range of tunability spans from 1.84 to 2 µm, while with the sesquioxides it can exceed 2.3 µm.

**Figure 2:**
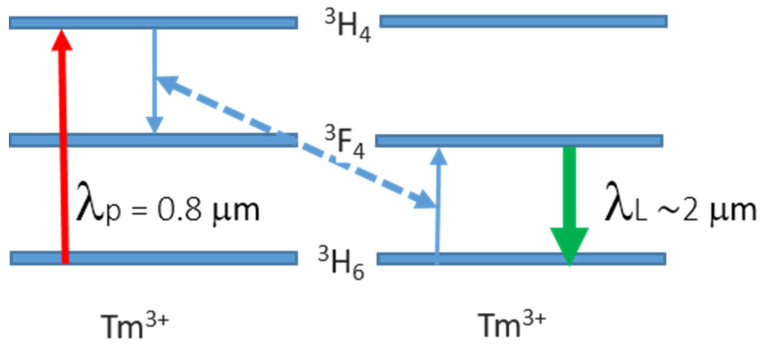
Scheme of the cross-relaxation (CR) process.

The concentration of Tm^3+^ ions is a crucial factor because, at high levels of doping cross-relaxation (CR) processes can take place between two neighboring Tm^3+^ ions [4,75], triggering the 2 µm transition, as is explained below (see Figure 2).

In the following sections we will discuss the properties of the main laser transitions and the conditions needed to activate them.

### 2.1. Tm^3+^ ^3^H_4_ → ^3^F_4_ Near-Infrared Emission at 1.5 µm

The existence of the ^3^H_4_ → ^3^F_4_ laser emission at around 1.5 µm [76,77,78,79] was demonstrated for the first time in 1983 in (Yb,Tm):BaYb_2_F_8_ and (Tm,Yb):LiYbF_4_ crystals pumped with a 1054 nm neodymium laser [80]. Five years later, laser oscillations in pure Tm^3+^-doped multimode fluoride fibers at around 1.48 µm, pumping with a krypton ion laser operating at 676 nm, were demonstrated [81]. Later on, other demonstrations followed in fibers [79,82]. Concerning its usage as a bulk material, few studies have been reported in the literature; the reason can be found in the nature of the transition, as it is a self-terminating four-level laser transition. As the lifetime of the lower laser level is longer than that of the upper state, laser oscillation occurs for a limited time, because the population accumulates in the lower laser level, so that eventually the population inversion cannot be maintained. This drawback is overcome by co-doping the matrices in order to either lengthen the upper level or shorten the lower level lifetime of the laser transition. It has been demonstrated that if hosts are co-doped with Tb^3+^ or Ho^3+^ ions and pumped at 0.6 or 0.8 µm, the lifetime of the ^3^H_4_ level can be shortened. Another possibility to depopulate the ^3^F_4_ is to exploit its excited level absorption toward the ^3^F_2_ level. This scheme was implemented by Komukai et al. [82] in ZBLAN glass fibers, using a Nd:YAG laser as a pump source. The excitation of Tm^3+^ to the ^3^H_4_ level was possible thanks to the weak absorption of Tm^3+^ in the ground state, in combination with the long absorption length allowed by the fibers. This effectively resulted in a two-photon pumping scheme. A similar scheme was proposed by Antipenko et al. [80] for crystals; in this case, the excitation of Tm was provided by absorption and multistep excitation transfer from Yb^3+^ ions used as sensitizers and pumped on the ^2^F_7/2_ → ^2^F_5/2_ transition. Concerning the nature of the host, essentially, when a sample doped with Tm^3+^ is pumped at 0.8 µm (i.e., ^3^H_6_ → ^3^H_4_), all radiative decay paths are activated. Fortunately, the mechanisms underpinning the laser’s action at different laser wavelengths are triggered differently. The usage of low-energy phonons together with low-doping materials is a good compromise; however, there is no shortage of exceptions. In CaF_2_, which is in principle an excellent large host with high thermal conductivity compared to other fluoride crystals and glasses, special attention is required. Due to the charge compensation [83] necessary to allow the neutrality of the host, even for low concentrations of dopant, aggregates of thulium ions can be formed, enhancing the probability of CR process triggering the transition at 2 µm. Fortunately, it has been demonstrated that in matrices co-doped with Y^2+^ ions it is possible to reduce the thulium aggregation. Thus far, no laser emission at this wavelength has been achieved in any laser ceramics.

### 2.2. Tm^3+^ ^3^F_4_ → ^3^H_6_ Near-Infrared Emission at 2 µm

The optical transition Tm^3+^ ^3^F_4_ → ^3^H_6_ [4] is widely used in laser systems with emission at around 1.9 µm. As it involves one of the thermally populated Stark levels of ^3^H_6_, emission from 1.8 to 2.2 µm can be obtained. In principle, the ^3^F_4_ → ^3^H_6_ optical transition can be excited by two different pumping schemes, which are characterized by different efficiencies. The least efficient scheme is based on direct pumping of the lower manifold ^3^H_6_ by a pump source at around 1.7–1.8 µm to the upper manifold ^3^F_4_ [5]; the most efficient is triggered by a non-radiative mechanism such as the cross-relaxation process (^3^H_6_ + ^3^H_4_ → ^3^F_4_ +^3^F_4_) in a matrix activated with a sufficient doping concentration [4,75], because the dipole–dipole interaction depends on the ion spacing, and shows a square dependence on the ion excitation density. If a pump wavelength centered at around 780 nm is used, Tm^3+^ ions are excited from the ^3^H_6_ to the ^3^H_4_ level. Thanks to the CR process, an electron of one ion relaxes from ^3^H_4_ to ^3^F_4_ (first excited ion), while an electron of the other ions is excited to ^3^F_4_ (second excited ion). Both ions contribute to populating the upper laser level ^3^F_4_ so that two excited ions are generated for each absorbed pump photon. This is referred to as a “*two-for-one cross-relaxation mechanism*”. As at room temperature a small percentage of the population (i.e., 2*at.*% in Tm:YAG) is placed on the high-energy states of the ground manifold, the ^3^F_4_→^3^H_6_ laser transition can be considered to be a quasi-three-level system whose overall quantum efficiency approaches 2 (beyond Stokes limit) [84], compensating for the high quantum defect. This transition can be activated in matrices with a level of doping higher than 2*at.*%, and moderates phonon energy. Historically, the first laser based on this transition with emission at 2 µm (Tm:YAG) was built in 1965 [85], while the pulsed regime was achieved 10 years later in a co-doped (Cr,Tm):YAG [34].

### 2.3. ^3^H_4_ → ^3^H_5_ Transition at 2.3 µm

The emission of Tm^3+^ ^3^H_4_ → ^3^H_5_ optical transition is centered at around 2.3 µm [34,86], and can be excited at 0.8 µm; it can be managed as a quasi-four-level laser transition, as non-radiative multiphonon relaxations depopulate the ^3^H_5_ level. However, its activation is complex, because the ^3^H_4_ level is a metastable state involved in several decay mechanisms. First, the ^3^H_4_ level is the upper level of the laser transition centered at 1.5 µm (i.e., ^3^H_4_ → ^3^F_4_); secondly, quenching mechanisms such as non-radiative relaxation, CR near Tm^3+^ ions, and energy transfer to impurities can depopulate the level. This oscillation was demonstrated in low-doping fluoride crystals [87,88,89], where non-radiative relaxation processes scarcely take place [90]. In YLF matrices, a continuous tunability was measured from 2.20 to 2.46 µm [91]. Lately, crystal oxides such as Tm-doped YAlO_3_ [92] and YAG [93] have been successfully tested. The dopant concentration underlying the CR mechanism between the ^3^H_4_ and ^3^F_4_ levels can be minimized, as several studies have already demonstrated, doping the host with a level of activators lower than 2*at.*%.

Unfortunately, to date, no laser oscillations at this wavelength have been demonstrated in ceramics.

### 2.4. Tm^3+^ Visible Laser Emissions

Although so far, to the best of our knowledge, no visible laser oscillations have been achieved and reported in the literature in mixed ceramics, it is interesting to observe that a Tm:YLF laser emitting in the blue, resonantly pumped by a XeF laser, was already presented in 1981 by Baer et al. [94]. Spectroscopic investigations focused on fluorophosphate glass hosts have been performed since 1984 and have demonstrated that the Tm^3+^ ^1^D_2_ → ^3^H_4_ transition can be used to obtain a laser oscillation in the visible region, at around 450 nm [95,96]. In fact, pumping of a 1*at.%* Tm:YLF crystal with two laser dyes at 780.78 nm and 648.77 nm oscillations in the blue (^1^D_2_ → ^3^F_4_ transition) and green (^1^D_2_ → ^3^H_5_ transition) regions was achieved; upconversion laser operation was at 77 K [97]. In 1992, blue upconversion laser emission at 450.2 nm (^1^D_2_ → ^3^F_4_ transition) and at 483.0 nm (^1^G_4_ → ^3^H_6_ transition) was also achieved in a Tm:YLiF_4_ crystal. The first transition was excited by sequential two-photon absorption with a Ti:sapphire laser source at 784.5 nm and 648 nm. In this experiment, the operation temperature was also up to 70 K. The laser emission at 483 nm was obtained by pumping with a single red dye laser whose wavelength was resonant with the absorption from a metastable intermediate state. The laser emission was observed up to 160 K [98]. In a (Tm,Yb):BaY_2_F_8_ crystal, where ytterbium plays the role of sensitizer, upconversion laser operation has been demonstrated in the visible region—in particular at 456 nm, 482 nm, 512 nm (Tm^3+^ ^1^D_2_ → ^3^H_4_ transition), and 649 nm. These measurements were performed at room temperature by pumping the sample with a CW 960 nm Ti:sapphire laser as well as a diode laser [99].

## 3. Mixed Ceramic Matrices

Before focusing on mixed-sesquioxide ceramics, some general consideration of ceramics may be useful for readers.

It has largely been demonstrated that transparent polycrystalline ceramics can overcome some drawbacks related to the melt growth of cubic sesquioxide crystals, which are characterized by high melting points (2490 °C in Lu_2_O_3_, 2485 °C in Sc_2_O_3_, 2425 °C in Y_2_O_3_) and by phase transition points lower than the melting points of the lattice [100].

Generally speaking, in ceramics it is possible to achieve high levels of doping and uniformity in dopant distribution, as well as excellent thermomechanical and optical properties comparable to their crystalline counterparts. Laser-grade ceramics can be fabricated thanks to the constant improvement in their manufacturing technique, because the mixture of powder fabrication methods and sintering processes plays a fundamental role in achieving the greatest optical transparency of ceramics. Today, ceramics with optical transparency near Fresnel’s theoretical limit are manufactured [101,102,103].

Nanocrystalline powders can be fabricated via several processes, such as combustion synthesis [104,105], the hydrothermal method [106], emulsion synthesis, sol–gel processing [107], co-precipitation in aqueous media [108,109,110], or laser ablation [111,112]. As far as the sintering process is concerned, the techniques currently being applied include vacuum sintering [113], vacuum sintering followed by hot isostatic pressing [114,115], hot pressing (HP) [116], hot pressing and hot isostatic pressing (HP–HIP) [117], spark plasma sintering (SPS) [118,119], and pressureless sintering under a flowing H_2_ atmosphere [120]. Of course, each production technique has strengths and weaknesses, imposing severe limitations on the dimensions and shape of the sample, its uniformity, and the fabrication time. For example, ceramics made by SPS can be fabricated in a short time; however, they have a small size. Moreover, the vacuum sintering temperatures of each material reported in the literature to date show a fairly narrow interval of variation, from 1700 °C to 1850 °C.

The mixed-sesquioxide ceramics mentioned in this review represent the solid solutions of the most common cubic sesquioxides, such as Lu_2_O_3_, Sc_2_O_3_, and Y_2_O_3_; they are obtained via a partial substitution of cations in the lattice by different rare-earth ions. For instance, (Sc_x_Y_1−x_)_2_O_3_ [53], (Lu_x_Y_1−x_)_2_O_3_ [51,52], and (Lu_x_Sc_1−x_)_2_O_3_ [54,55,56] matrices can be obtained by replacing an Y^3+^ ion with Sc^3+^ or Lu^3+^ in the first two hosts, and a Sc^3+^ ion with Lu^3+^ in the last matrix.

A major challenge in the process of lasing quality mixed-sesquioxide ceramics is the attainment of a pore-free microstructure with homogeneous distribution of chemical components at the nanoscale level, so as to avoid refractive index modulation near grain boundaries. In this regard, two main fabrication methods can be distinguished: The first method consists of a sintering plus HIP approach, with the utilization of commercially available powders or nanoparticles synthesized by co-precipitation and added ZrO_2_ as a grain growth inhibitor [54,121,122]. The second method involves conventional vacuum sintering of weakly agglomerated nanoparticles produced by laser ablation [53,112].

The main effect of substitutions, due to the different masses and radii of the cations (i.e., r_ion_(Y^3+^) = 0.9 Å, r_ion_(Sc^3+^) = 0.745 Å and r_ion_(Lu^3+^) = 0.861 Å [49]), is a change in the lattice parameters because the size of the unit cell increases and, in turn, modifies the crystal field strength; the latter affects the position and the linewidth of the Tm^3+^ levels. The large splitting of the levels results in a broad range of tunability—broader than in pure matrices—while the emission spectra assume a smoother shape, better suited for a mode-locking regime. It was clearly demonstrated in Tm^3+^-doped (Sc_x_Y_1−x_)_2_O_3_ that the decrease in the cation radius increases the crystal field strength and, thus, the Stark splitting of manifolds involved in such laser transitions as, for instance, ^3^F_4_, ^3^H_6_, and so on [123]. At the same time, the separation between the lowest Stark electronic sublevels of the two manifolds ^3^F_4_ and ^3^H_6_ remains nearly identical when moving from Tm:Y_2_O_3_ to Tm:Sc_2_O_3_.

Concerning the crystallographic properties, yttrium, lutetium, and scandium sesquioxides are isostructural, and are capable of forming continuous series of solid solutions (Lu_x_Y_1−x_)_2_O_3_, (Sc_x_Y_1−x_)_2_O_3_, and (Lu_x_Sc_1−x_)_2_O_3_, with a bixbyite-type cubic structure (space group Ia3–Th7). However, when the difference in the ionic radii of the substituting and substituted cations is significant, crystallization in a different, completely ordered lattice (i.e., perovskite structure) can be observed. For example, at room temperature and pressure the smallest rare-earth ion Sc^3+^ and several lanthanides (Ln, from La^3+^ to Ho^3+^) form Ln-scandates—orthorhombic perovskites with the space group Pbnm [124]. In addition, pure perovskite-type YScO_3_ phases have previously been synthesized via the flux method using a PbO/PbF_2_ flux [125], by a combination of the polymerized complex method and solid-state reaction [126] and by heating up a corresponding mixture of oxides to 1000 °C at 20 kbar for 1 h [127]. Conversely, only a bixbyite-type cubic structure was detected in polycrystalline samples based on mixed (Lu,Y,Sc)_2_O_3_ matrices fabricated using ceramic technology.

### 3.1. Thermal Conductivity of Mixed Ceramics

As already mentioned, the thermal conductivity (K) of laser gain matrices depends on two parameters: the nature of the host, and the concentration of the dopant. Garnets, for instance, have a higher undoped thermal conductivity than fluorides, but lower than that of the sesquioxides, which are preferred for the development of high-average-power laser systems [128]. In all matrices, a decrease in thermal conductivity is observed with increased dopant concentration [50]. This phenomenon can be attributed to the different weight of the substituted cation and active RE ion, because the phonons—which transport the heating in insulator materials—are scattered at the doping ions, also known as mass defects, with a consequent decrease in K [129]. Accordingly, only Lu^3+^-based hosts do not show this trend, because the mass of Tm^3+^ is similar to that of Lu^3+^.

In undoped mixed matrices, the thermal conductivity is expected to be lower than in pure matrices due to the intrinsic disorder; moreover, it decreases when increasing the concentration of dopants. This has been very well demonstrated in mixed crystals such as LuScO_3_ [130]. We have no theoretical reasons to suppose that this should be different in mixed matrices; on the contrary, this could explain the saturation measured, for instance, in CW Tm^3+^:LuYO_3_ ceramic emissions [51].

As the data on thermal conductivity of mixed ceramics and its temperature dependence are scarce or nonexistent, only the values for several RE^3+^-doped disordered sesquioxide crystals can be reported. For instance, Liu et al. measured 5.488 W/(m K) and 5.019 W/(m K) for 1*at.*% Yb:Lu_0.99_Y_1.01_O_3_ and 1*at.*% Yb:LuScO_3_ crystals, respectively [131]. Krankel et al. reported Czochralski growth and temperature-dependent thermal conductivity of (Er_0.07_Sc_0.50_Y_0.43_)_2_O_3_ crystals [132]. At room temperature the thermal conductivity reached 4.1 W/(m K). Nevertheless, these values are comparable with those of other disordered matrices, such as RE^3+^-doped CaGdAlO_4_ and Ca_3_Nb_1.5_Ga_3.5_O_12_ [133,134], exhibiting broad gain bandwidth suitable for ultrashort-pulse laser operation.

### 3.2. Description and Comparison of the Fabrication Techniques Used to Develop the Mixed Sesquioxides Ceramics

Recently, an alternative method of synthesizing sesquioxide transparent ceramics has been proposed—to reduce the sintering temperature by forming a solid solution [46,135]. It was found that Y_2_O_3_, Sc_2_O_3_, and Lu_2_O_3_ can be mixed in arbitrary ratios to form a complete solid solution series with the formula of (Lu_x_Y_y_Sc_1−x−y_)_2_O_3_ (0 ≤ x < 1, 0 ≤ y < 1, and 0 < x + y ≤ 1), and the melting points of the binary and ternary systems are generally lower than that of any constituent component [136,137].

It is essential for laser applications to fabricate highly transparent sesquioxide ceramics with clean grain boundaries and narrowly distributed grain size. The availability of powders with desired properties, such as good dispersion and high reactivity, is crucial to obtaining better quality ceramics, because large particle size and hard aggregation may necessitate high sintering temperatures and, thus, may cause exaggerated grain growth [46].

As for the sintering methods, the solid-state reaction method is one of the conventional ways to sinter powders for polycrystalline transparent ceramics. This method is often used for the fabrication of complex oxides (such as YAG [7] starting from Y_2_O_3_ and Al_2_O_3_ powders, or LuAG from Lu_2_O_3_ and Al_2_O_3_ powders [31]), where the formation of the final complex oxide occurs during the sintering phase. Another conventional method is non-reactive sintering of nanopowders with high sinterability. In this kind of process, the starting powders already have their final chemical composition, and in the sintering phase the ceramic is formed by growth and densification of the microcrystals. The starting powders are usually obtained via wet chemical reactions, including homogeneous precipitation [138], the sol–gel method [139], co-precipitation [55,121,140,141,142] methods, etc. Other investigations have addressed the use of spark plasma sintering (SPS) for the production of Sc_2_O_3_, Y_2_O_3_, and Lu_2_O_3_ transparent ceramics [118,119], but not yet for the production of mixed sesquioxides.

### 3.3. Tm^3+^: (Lu_1−x_Sc_x_)_2_O_3_ with 0 < x < 1

In 2017, for the first time, a Tm^3+^:LuScO_3_ ceramic was fabricated by using a solid-state reactive sintering method with an average grain size of 1.65 µm [55]. The laser behavior of the sample was tested by CW pumping the sample at 790 with an AlGaAs laser diode, obtaining an output power of 211 mW with slope efficiency of ~8.2%. The mode-locking regime was demonstrated using single-walled carbon nanotubes (SWCNTs) as a saturable absorber. The shortest pulse width was 0.59 µs, with a repetition rate of 34.72 kHz. The maximum average output power was 32 mW. Concerning the spectroscopic data, and from comparison of spectra obtained for crystals and ceramics, no relevant differences in peak location were observed. The room temperature absorption spectrum of ceramic samples from 300 to 2100 nm shows six bands associated with Tm^3+^ transitions from the ^3^H_6_ ground state to ^1^D_2_, ^1^G_4_, ^3^F_2,3_, ^3^H_4_, ^3^H_5_, and ^3^F_4_ excited states, respectively. The peak absorption cross-section and its full width at half-maximum (FWHM) for the Tm^3+^ ^3^H_6_ → ^3^H_4_ transition at 793 nm were calculated to be 3.5 × 10^−21^ cm^2^ and 32 nm, respectively. The absorption cross-section is comparable to that of Tm^3+^:Lu_2_O_3_ ceramic and crystal (3.8 × 10^−21^ cm^2^ at 796 nm). The emission spectrum covers the wavelength range from 1550 to 2200 nm and corresponds to the transition ^3^F_4_ → ^3^H_6_ of the Tm^3+^ ion. The emission band centered at 1970 nm measures an FWHM of 75 nm and stays between the peaks measured in Sc_2_O_3_ at 1940 nm and in Lu_2_O_3_ at 1980 nm [143,144]. The multipeak structure measured in pure matrices disappears in mixed ceramics and crystals due to the spectral broadening generated by the disorder induced by the substitution of Lu^3+^ ions with Sc^3+^ ions. These ions are characterized by different masses and radii, as explained in the previous section; additionally, the latter exhibits smoothing of the whole spectrum, which could be helpful in obtaining ultrashort-pulse lasers via the mode-locking technique. Concerning the fluorescence lifetime of the ^3^F_4_ level, it was measured to be 3.2 ms, which is comparable with that of Tm^3+^:Lu_2_O_3_ ceramic (i.e., 3.7 ms [16]) and Tm^3+^:Lu_2_O_3_ crystal (i.e., 3.8 ms [143,144]). The large emission band and long lifetime clearly indicate that Tm^3+^:LuScO_3_ ceramic is a very promising material in mode-locking regimes.

The same year, an excellent result was obtained when a 4.76*at.*% Tm^3+^:(Lu_2/3_Sc_1/3_)_2_O_3_ mixed-sesquioxide ceramic [121] was synthesized via hot isostatic pressing (HIP), improving upon the results reported in [55]. According to the absorption spectrum data acquired at room temperature, the absorption cross-sections were calculated for the transitions ^3^H_6_ → ^3^F_4_ and ^3^H_6_ → ^3^H_4_, finding σ_abs_ = 4.26 × 10^−21^ cm^2^ at 1622 nm and σ_abs_ = 2.80 × 10^−21^ cm^2^ at 793 nm (the FWHM was 25 nm). The broadening of the emission peaks with respect to the pure matrices (i.e., Lu_2_O_3_, Sc_2_O_3_) was confirmed at 6 K as well. Spontaneous radiative transitions and radiative lifetimes were assessed by means of the Judd–Ofelt theory. The probabilities for the maximum spontaneous radiative transitions, σ_SE_, were σ_SE_ = 7.15 × 10^−21^ cm^2^ at 1951 nm and σ_SE_ = 2.38 × 10^−21^ cm^2^ at 2090 nm. Concerning the radiative lifetime of the lowest excited state, it was found that τ_rad_(^3^F_4_) = 4.01 ms, which is comparable with the values of the luminescence time measured in 1*at.*% Tm:LuScO_3_ single crystal and 2*at.*% Tm:LuScO_3_ ceramic, i.e., 3.84 and 3.2 ms, respectively. The laser behavior in the CW regime was tested by a microchip-type cavity. The maximum output power was 1.01 W at 2095 and 2102 nm, with a slope efficiency of η = 24% for T_OC_ = 3%. The laser threshold was P_abs_ = 0.86 W. In the mode-locking regime obtained via a near-surface design GaSb-based SESAM, it was demonstrated that Tm^3+^:(Lu_2/3_Sc_1/3_)_2_O_3_ mixed ceramics can generate nearly Fourier-limited pulses of 63 fs pulse duration. The sample was pumped with a high-beam-quality Ti:sapphire laser source.

Mixed-sesquioxide ceramics with different Lu/Sc balances were fabricated via the solid-state reactive sintering method (pre-sintered in vacuum and then post-sintered by HIP) using ZrO_2_ (99.5%) powder as a sintering aid, i.e., 1*at.*% and 1.5*at.*% Tm:Lu_1.6_Sc_0.4_O_3_ with a thickness of 2.6 mm [54]. The high optical quality of these ceramics, which have an average grain size of 1.54 µm, was demonstrated by transmittance measurements, i.e., 82.21% at the lasing wavelength of 2.09 µm, which is near to the Fresnel theoretical value of 82.28% (*n* = 1.906 at 2 µm). The samples were excited by a fiber-coupled AlGaAs diode laser at 796 nm in order to measure the emission spectra at room temperature and 77 K, and these results were compared in order to calculate the Stark splitting of the levels involved in laser action: a Stark splitting of 920 cm^−1^ in the ground state ^3^H_4_ and 472 cm^−1^ in the ^3^F_4_ of Tm^3+^ was found. Thanks to these large Stark splittings, the laser can emit at around 2.09 µm due to transition from the lowest Stark level ^3^F_4_ to the highest Stark level of ^3^H_6_. The stimulated emission cross -section at 2.09 µm is σ_em_ = 1.1 × 10^−21^ cm^2^. From the absorption spectra, the absorption cross-section at 796 nm responsible for the ^3^H_6_ → ^3^H_4_ transition was calculated, finding it to be σ_abs_ = 3.1 × 10^−21^ cm^2^. A plano-concave cavity was used to test the samples; for the 1*at.*% Tm^3+^ ceramic, the maximum output power was 9.8 W, with a slope efficiency of 40%; the optical-to-optical conversion efficiency was measured at 25.8%. Comparable results were found with the 1.5*at.*% Tm^3+^ laser, i.e., 11 W with 39% slope efficiency and an optical-to-optical efficiency of 28.9%. With both samples, the emission wavelength was 2090 nm. By comparing the theoretical data (38%) for the conversion from one 796 nm photon to one 2090 nm photon, it was clear that cross-relaxation between two closed Tm^3+^ ions took place.

Over the years, various manufacturing techniques have been developed to improve the optical qualities of ceramics. Recently, a 2*at.*% Tm^3+^:(Lu_0.8_Sc_0.2_)_2_O_3_ was obtained via the gel-casting of well-dispersed nanopowders obtained by co-precipitation method using an alcohol–water solvent, which was expected to yield more homogeneous and transparent ceramics [122]. The excellent optical quality of samples was carried out from the optical in-line transmittance of a 12 mm long ceramic rod, with results as high as 80.3% at 2090 nm, which is comparable with the theoretical transmittance of ~81.4% at 2090 nm. The average grain sizes spanned from 2 to 5 µm. The attenuation coefficient at 2090 nm was 0.006 cm^−1^. The absorption and emission spectra of the sample were acquired at room temperature. Concerning the absorption bands, four bands centered at 686, 796, 1207, and 1625 nm were attributed to the Tm^3+^ transitions from its ground state of ^3^H_6_ to the excited states of ^3^F_2,3_, ^3^H_4_, ^3^H_5_, and ^4^F_4_, respectively. Focusing on the whole ^3^H_6_→^3^H_4_ absorption band that peaked at 796 nm, its FWHM was around 38 nm, which is broader than that of Tm^3+^-doped Lu_2_O_3_ ceramics. Accordingly, in the CW laser test, the sample, having been placed in a plano-concave cavity, was pumped using a commercial laser diode with emission at 796 nm. A maximum output power of 1.88 W was measured, with a slope efficiency of 24.6% (with respect to the input pump power) and an optical-to-optical efficiency of 14.1%. The laser threshold of the absorbed pump power was 3.2 W.

Recently, a 58 fs laser pulse was generated at 2081 nm by Kerr-lens mode locking in a 2.8*at.*% Tm:(Lu_2/3_Sc_1/3_)_2_O_3_ transparent ceramic [56] fabricated by hot isostatic pressing (HIP) of commercial powders at 1800 °C and 195 MPa in an Ar atmosphere [121]. In CW operation mode, this delivers a maximum output power of 490 mW at around 2088 nm (T_OC_ = 0.5%), with an efficiency conversion of 26.8%. Short pulses were obtained in a Kerr-lens mode-locking regime by exploiting the high nonlinear refractive index (n_2_) of the ceramic. We should note that in Tm:Lu_2_O_3_ ceramics this was measured at n_2_ = 3.3 × 10^−16^ cm^2^/W at 2070 nm [15] and n_2_ = 8.6 × 10^−16^ cm^2^/W at 1064 nm, and it is expected that in mixed-sesquioxide ceramics this value should be almost preserved. The average output power was 220 mW at a pulse repetition rate of 84.8 MHz. The peak on-axis laser intensity in the sample was ∼510 GW/cm^2^. It was very well demonstrated that the emitted spectra at wavelengths longer than 2.2 µm are attributable to vibronic transitions of the Tm^3+^ and played a fundamental role in achieving laser pulses as short as 58 fs.

For the sake of clarity and completeness, we should recall that in 2011 the first 1*at.*% Tm^3+^-doped LuScO_3_ crystal was grown by Koopmann et al. [143]. In CW, an output power of 705 mW with a slope of 55% at around 2.1 µm was measured in a nearly concentric resonator; the laser threshold was only 0.38 mW. Concerning the range of tunability, it was demonstrated from 1960 to 2115 nm (i.e., 155 nm). Two years later, a Ti:sapphire-pumped mode-locked Tm:LuScO_3_ crystal laser was demonstrated [145]. In 2018, a new high-optical-quality mixed-sesquioxide crystal—i.e., 4*at.*% Tm:LuScO_3_—was grown via the heat exchanger method in a rhenium crucible and tested in a four-mirror z-fold cavity pumping for the first time by a laser diode at 793 nm [146]. In a CW regime, the laser delivered 660 mW at 2102 nm (T_OC_ = 2%), with a slope efficiency of up to 33%, while the laser threshold was 194 mW. The tunability of laser was measured from 1973 nm to 2141 nm (i.e., 148 nm), with an FWHM of 75 nm. The femtosecond mode-locking operation was achieved via an ion-implanted InGaAsSb quantum-well-based SESAM, which enabled the generation of near-transform-limited pulses of 170 fs at 2093 nm, with an average output power of 113 mW and a pulse repetition frequency of 115.2 MHz.

### 3.4. Tm^3+^: (Lu_1−x_Y_x_)_2_O_3_ with 0 < x < 1

The year 2017 was a profitable one, as the first 3*at.*% Tm-doped (Lu_0.5_Y_0.5_)_2_O_3_ was also fabricated by solid-state reactive sintering (T_sintering_ = 1700 °C) and tested in CW and Q-switching regimes [51]. The sample was pumped with a fiber-coupled AlGaAs laser diode at 790 nm. In CW, it delivered a maximum output power of 1.55 W, with a slope efficiency of 19.9% at 2050 nm. Passive Q-switching was obtained using a Cr:ZnSe saturable absorber. At 2047 nm, a maximum average output power of 0.54 W was found, along with a pulse width of 120.3 ns and a pulse energy of 20.5 µJ, while the pulse peak power was 170.6 W. Spectroscopic measurements have shown that the location of the main absorption peaks at 775.5, 796.5, and 811.5 nm matched those of Tm-doped Lu_2_O_3_ [16] and Y_2_O_3_ ceramics [25], and the calculated absorption coefficient at 796.5 nm and 790 nm was 2.6 cm^−1^ and 1.6 cm^−1^, respectively.

Two years later, the first mode-locking regime was demonstrated with a 3*at.*% Tm:LuYO_3_ mixed ceramic synthesized via a solid-state reactive sintering method [147]. The mode-locking laser operation was achieved by inserting an SESAM with a modulation depth of 1.2% and a relaxation time of 10 ps into the cavity. Pulses with a duration of 41 ps and a repetition rate of 139.3 MHz at 2061 nm were obtained. Concerning the spectroscopic measurements, the sample was pumped with a fiber-coupled laser diode at 796 nm. The absorption cross-section at the pump wavelength was calculated to be σ_abs_ = 3.8 × 10^−21^ cm^2^, with an FWHM of 25 nm. This is comparable with results obtained in Tm:Lu_2_O_3_, and higher than in Tm:LuScO_3_, where σ_abs_ = 3.5 × 10^−21^ cm^2^ was found [55]. Conversely, emission cross-sections calculated at the two main peaks of the fluorescence spectra—i.e., at 1937 nm and at 2055 nm—were found to be σ_em_ = 6.0 × 10^−21^ cm^2^ and 3.0 × 10^−21^ cm^2^, respectively; these results are lower than in 2*at.*% Tm^3+^:Lu_2_O_3_ (i.e., 8.2 × 10^−21^ cm^2^ [16]) and Y_2_O_3_ (i.e., 9.9 × 10^−21^ cm^2^ [148]). The fluorescence time of the ^3^F_4_ → ^3^H_6_ laser transition was 2.6 ms. CW laser behavior was tested by closing the cavity using output couplers with different transmissions (T_OC_). The maximum output power of 1.20 W, with a slope efficiency of 25.1% and threshold of 0.53 W, was obtained with a T_OC_ = 3%. The highest slope efficiency of 33.1% was achieved with a T_OC_ = 10%, with a maximum output power of 0.88 W and threshold of 1.5 W.

Passive mode-locking of a 3*at.*% Tm:LuYO_3_ ceramic was also demonstrated by using a single-walled carbon nanotube saturable absorber [59]. When pumping at 795 nm with a Ti:sapphire laser, pulses as short as 57 fs were measured at 2045 nm, with an average power of 63 mW and a repetition rate of 72.6 MHz. This is an excellent achievement compared with those obtained in a Tm:Lu_2_O_3_ ceramic operating in a mode-locking regime with a saturable absorber, i.e., 180 fs [15]; it is similar to the pulse duration of 63 fs obtained with Lu_2/3_Sc_1/3_O_3_ [54]. The tunability of the sample in the CW regime was preliminarily tested by inserting a 3.2 mm thick birefringent quartz plate into the cavity. The curve of tunability ranged from 1909 to 2109 nm—i.e., 200 nm—with the main peak located at 2074.2 nm and a corresponding maximum output power of 440 mW (T_OC_ = 1.5%). Under the same experimental conditions, in 3*at.*% Tm:Lu_2/3_Sc_1/3_O_3_ ceramic, a tuning range as broad as 130 nm was found [54].

Recently, the same group has measured the shortest pulses [52,149] ever reported in literature for any bulk solid-state lasers based on Tm^3+^. The gain material was a 3*at.*% Tm:LuYO_3_ ceramic mode-locked with a GaSb-based SESAM. At a repetition rate of ∼78 MHz, a pulse of 54 fs was measured at 2049 nm, with an average output power of 51 mW.

The laser performances of a ceramic fabricated by hot isostatic pressing with a sintering temperature of 1700 °C were tested in CW, pumping the ceramic with a continuous-wave narrow-line-bandwidth Ti:sapphire laser at 795.3 nm. By closing the four-mirror bow-tie cavity using an output coupler with a transmission of T_OC_ = 3%, an output power of 603 mW with a slope efficiency of 33.2% and a laser threshold of 200 mW was measured.

Concerning Tm:LuYO_3_ crystals, a sample was grown via the optical floating zone method for the first time in 2020 [150]; unfortunately, no data on laser behavior have been reported in the literature. Spectroscopic characterization at room temperature was performed, finding an absorption cross-section at 796 nm of σ_abs_ = 7.3 × 10^−20^ cm^2^ with the FWHM of 48 nm; lower values were measured at 2057 nm (σ_abs_ = 0.2 × 10^−20^ cm^2^) and 1936 nm (σ_abs_ = 1.4 × 10^−20^ cm^2^). The emission cross-section at 2056 nm was σ_em_ = 1.95 × 10^−20^ cm^2^, with an FWHM of nearly 300 nm. The measured fluorescence lifetime of ^3^F_4_ was 0.86 ms.

### 3.5. Tm^3+^: (Y_1−x_Sc_x_)_2_O_3_ with 0 < x < 1

(Y,Sc)_2_O_3_ is very attractive because the large difference in the ionic radii of Y^3+^ and Sc^3+^ [49] is expected to enhance the disorder of the lattice and, in turn, a large Stark splitting of Tm^3+^ levels should be measured. However, it has been extremely difficult to grow large, single crystals, due to a phase transition from hexagonal to cubic structure occurring at a temperature near to the melting point of yttrium oxide, above 2400 °C. However, thanks to the continued efforts of the scientific community, small matrices doped with Nd^3+^, Er^3+^, and Yb^3+^ have been grown and investigated. The first Nd^3+^:YScO_3_ crystal with a cubic bixbyite-type structure was grown in 1976 [151]. An investigation of the excitation spectra and fluorescence dynamics of Er^3+^:YScO_3_ single-crystal fiber grown via the laser-heated pedestal growth (LHPG) method was reported in 1991 [152]. The orthorhombic perovskite single-crystal Y_0.96_ScO_2.94_ grown from mixtures of sesquioxides with the flux reagents PbF_2_ and PbO was spectroscopically investigated, and nuclear magnetic resonance of a sample doped with 1*at.*% Yb^3+^ was carried out [125]. X-ray diffraction analysis and micro-Raman characterization were carried out in a 0.1*at.*% Nd^3+^:YScO_3_ crystal fiber grown via the LHPG method [153].

In 2013, for the first time, Yb^3+^-doped (Sc_x_Y_1−x_)_2_O_3_ ceramics with x = 0.1 and 0.9, developed using high-purity nanopowders and pressureless sintering in a H_2_ atmosphere, were fabricated [154,155]. The first laser oscillation was demonstrated only recently in 6*at.*% Yb-doped (Sc*_x_*Y_1−x_)_2_O_3_ ceramics with different Y^3+^/Sc^3+^ balances (x = 0, 0.273, 0.508, and 0.742), fabricated via solid-state vacuum sintering of laser-ablated mixed-sesquioxide nanoparticles [112].

To the best of our knowledge, only one study focused on Tm^3+^:(Y_1−x_Sc_x_)_2_O_3_ with 0 < x < 1 ceramic has been reported in the literature [53,156]. This study demonstrated the first laser action of a 5*at.*% Tm^3+^-doped (Sc_0.252_Y_0.698_)_2_O_3_ ceramic made via solid-state vacuum sintering of laser-ablated mixed-sesquioxide nanoparticles. Pumped at 793 nm in quasi-CW mode, this ceramic delivers 1.24 W with a slope efficiency of 9.45% at 2076 nm. Absorption spectra were acquired at several temperatures, from 93 K to 293 K: three broad bands located at 670–700 nm, 760–820 nm, and 1600–1700 nm were observed, associated with Tm^3+^ transitions from ^3^H_6_. The variation of the temperature affects the absorption coefficients (α) in different ways. In the case of the main peaks (657, 685, 764, and 774 nm), the α-coefficient decreases when increasing the temperature; conversely, the peaks located at 697 nm as well as in the range of 779–840 nm show an enhancement of α. This can be attributed to the change in the thermal population distribution of the sublevels in the ^3^H_6_ manifold. When increasing the temperature, the population in the upper sublevels increases, enhancing the absorption from the transitions starting from the upper sublevels, and the population in the lowest sublevel decreases, reducing the absorption coefficient of the transition starting from this sublevel. It is interesting to observe that when compared with Tm:Y_2_O_3_ the well-defined peak structure observable in the Tm:Y_2_O_3_ spectra is almost lost in the disordered sample—a clear signature of the disorder of the lattice. The individual sublevels of the ^3^H_6_ ground manifold are inhomogeneously broadened by the variation in the Stark splitting occurring at different lattice sites due to the random partial substitution of Y^3+^ cations with smaller Sc^3+^ ions. In mixed (Sc_0.252_Y_0.698_)_2_O_3_ the main peaks are less intense and broader; moreover, they are shifted to shorter wavelengths. In particular, the absorption coefficient (α) at 685.3 nm decreases from α = 140.6 cm^−1^ at 93 K to α = 81.9 cm^−1^ at room temperature. In Tm:Y_2_O_3_, the measured values were α = 79.8 cm^−1^ at 93 K and α = 66.1 cm^−1^ at RT. Regarding the emission spectrum, (Sc_0.252_Y_0.698_)_2_O_3_ shows two peaks located at 1946 nm and 2098 nm. If compared with Tm:Y_2_O_3_ [53], it can be observed that the emission cross-section at 1946 nm decreases from σ_em_ = 10.4 × 10^−21^ cm^2^ in Tm:Y_2_O_3_ to σ_em_ = 7.6 × 10^−21^ cm^2^ in the disordered matrix (Sc_0.252_Y_0.698_)_2_O_3_. However, the peak at a longer wavelength is shifted from 2060 nm (Tm:Y_2_O_3_) to 2098 nm (Sc_0.252_Y_0.698_)_2_O_3_. The emission cross-section at 2060 nm in Tm:Y_2_O_3_ was σ_em_ = 5.1 × 10^−21^ cm^2^, while σ_em_ = 3.9 × 10^−21^ cm^2^ at 2098 nm was found in (Sc_0.252_Y_0.698_)_2_O_3_. The CW tuning range was investigated in a coupled cavity configuration with an external grating, with 600 lines/mm placed in the Littrow configuration—a continuous range of tunability from 1927.5 nm to 2108.5 nm, i.e., 181 nm was measured.

## 4. Discussion

The continuous improvement of ceramic fabrication techniques has made it possible to obtain samples with high optical quality as well as materials that are difficult to produce in crystalline form, such as lutetia, for laser applications. Thanks to these ceramics, various research groups have developed lasers with output powers of hundreds of watts and short pulses. Without any doubt, today, the laser performance achieved by transparent polycrystalline ceramics is almost comparable with the corresponding crystals. Ceramic matrices as YAG, LuAG, LuYAG, sesquioxides, etc., doped with Tm^3+^ ions, have been spectroscopically tested, and laser oscillations have been achieved in the so-called *eye-safe region* (above 1.4 µm).

In the last five years, alongside the interest in the most common matrices [157,158], the idea of manufacturing mixed matrices (see Table 1 and Table 2) doped with various rare-earth ions [159,160] has emerged because, due to their crystalline structure distorted by the replacement of some cations of the matrix with other rare-earth ions, they could pave the way for the development of lasers with pulses in the order of a few tens of femtoseconds. The first results published in the literature are encouraging, as 50 fs laser pulses have been demonstrated (see Table 3). However, the knowledge we have on the spectroscopic behavior of this mixed matrix is limited to some hosts that have already been fabricated, and the physical and thermomechanical parameters are not yet known. Accordingly, a great effort will have to be made in terms of improving both the quality of the mixed matrices and the knowledge of the physical processes that determine the activation of some specific laser transitions. For instance, we believe that the development of lasers with visible emission operating at room temperature could be one of the most important goals.

The spectroscopic properties and laser behavior (i.e., laser emission wavelength, tuning range, and available pulse durations) of sesquioxide solid solutions are directly dependent on the composition of the mixed matrix, and the selection of a particular balance between cations should be based on the specific field in which the active medium and corresponding laser are planned to be applied. For instance, compared to (Lu_x_Y_1−x_)_2_O_3_ materials, mixed compositions with Sc—such as (Lu_x_Sc_x_)_2_O_3_ and (Y_x_Sc_x_)_2_O_3_—possess broader absorption and emission spectra of Tm^3+^ ions, supporting an extended tuning range and shorter pulse durations at the expense of a somewhat lower thermal conductivity, due to the larger difference in ionic radii and mass between Lu^3+^ (Y^3+^) and Sc^3+^. For enhanced Tm^3+^-doping concentrations and high-average-power laser action, disordered ceramics and crystals based on Lu_2_O_3_ seem more preferable, owing to the small discrepancy between Lu^3+^ and Tm^3+^, inducing a minor level of disorder and better preserving the thermophysical characteristics.

Considering possible mass production of mixed-sesquioxide gain media, the cost of raw materials should be taken into account. High-purity Y_2_O_3_ micro- and nanopowders are currently available on the market at a reasonable cost, while proper lutetium and scandium sesquioxide powders are much harder to find, and they are several times more expensive than yttria. Even though the modern fabrication methods yield disordered ceramic matrices with lasing quality, considerable efforts will be required for obtaining large-sized and large-aperture active components, so as to give an impulse to further development of the advanced 2 μm solid-state lasers required in various fields of science and technology. In this regard, the approach suggested by Wu et al., consisting of gel-casting of well-dispersed nanopowders followed by HIP treatment [122] for the synthesis of 12 mm long 2*at.*% Tm:(Lu_0.8_Sc_0.2_)_2_O_3_ ceramic rods, with an attenuation coefficient of ~0.006 cm^−1^ at 2090 nm, is a major step towards large, high-optical-quality samples.

Tm^3+^-doped mixed-sesquioxide materials could be elaborated in the direction of additional broadening of gain profiles by the utilization of Ho^3+^ as a co-dopant. The point is that the gain profiles of Tm^3+^ and Ho^3+^ in co-doped hosts have overlapping regions, and it is possible to combine stimulated emissions of both active ions for ultrashort-pulse lasing. For instance, Wang et al. [44] demonstrated 46 fs pulses at 2033 nm from an SESAM mode-locked Tm,Ho:Ca(Gd,Lu)AlO_4_ laser, which is the shortest duration ever obtained in a Tm- and/or Ho-based gain medium. The aspect of optimal ratio between Tm and Ho ions in sesquioxides—and especially in mixed sesquioxides—needs detailed investigation.

## 5. Conclusions

In this paper, we report a summary of fabrication techniques, spectroscopy, and laser performance of trivalent thulium-doped mixed-sesquioxide ceramics, i.e., (Lu,Sc,Y)_2_O_3_. In particular, a careful description of the properties and mechanisms of the thulium laser transition emission at around 1.5, 1.9, and 2.3 µm is given. The results obtained with Tm^3+^-doped ceramic hosts were compared to performance achieved with the corresponding crystals in all pumping regimes. The emission wavelengths ranging from 1.5 µm up to 2.3 µm make the trivalent thulium ions very useful, as Tm^3+^ can be used in a consistent number of applications, from medicine to LIDAR (light detection and ranging) tests. Although important milestones have already been achieved by thulium-based lasers, their features are still unexplored in fields such as, for instance, laser inertial fusion energy.

## Figures and Tables

**Table 1:** Spectroscopic data on the mixed ceramics reported in the literature; λ_abs_ and λ_em_ are the wavelengths at which the absorption and emission cross-sections were calculated, respectively.

Sample	Doping *at.*%	σ_abs_ (×10^−21^ cm^−2^)	λ_abs_ (nm)	σ_em_ (×10^−21^ cm^−2^)	λ_em_ (nm)	Grain Size (µm)	Lattice Const. (Å)	Ref.
LuScO_3_	2	3.5	793	-		1.65		[55]
(Lu_2/3_Sc_1/3_)_2_O_3_	4.76	2.8	793	7.15	1951	4–5	10.3683	[121]
(Lu_2/3_Sc_1/3_)_2_O_3_	4.76	4.2	1622	2.38	2090			[121]
Lu_1.6_Sc_0.4_O_3_	1.5	3.1	796	1.11	2090	1.54		[54]
Lu_0.8_Sc_0.2_O_3_	2	-		-		2.5		[122]
(Lu_2/3_Sc_1/3_)_2_O_3_	2.8	-		7.0	1950	-	-	[56]
LuYO_3_	3	3.8	796	6.0	1937	1.65	-	[51]
LuYO_3_	3	3.0	2055	-		1.65	-	[147]
LuScO_3_ crystal	1	2.6	793	8.0	1956	-	10.105	[143]
(Sc_1/4_Y_3/4_)_2_O_3_	5			3.9	2098	28.2	10.401	[53]

**Table 2:** CW and *quasi*-CW laser performance of the mixed ceramics reported in the literature; P_out_: laser output power; λ_L_: laser wavelength emission; η: slope efficiency; P_th_: laser threshold.

Sample	Doping *at.*%	P_out_ (W)	λ_L_ (nm)	η (%)	P_th_ (W)	Ref.
LuScO_3_	2	0.211	1982	8.2	0.840	[55]
(Lu_2/3_Sc_1/3_)_2_O_3_	4.76	1	2100	24	0.860	[121]
Lu_1.6_Sc_0.4_O_3_	1	9.8	2090	40	2.8	[54]
Lu_1.6_Sc_0.4_O_3_	1.5	11	2090	39	5.0	[54]
Lu_0.8_Sc_0.2_O_3_	2	1.88	2090	24.6	3.2	[122]
(Lu_2/3_Sc_1/3_)_2_O_3_	2.8	0.490	2088	26.8	-	[56]
LuYO_3_	3	1.55	2050	19.9	1.1	[51]
LuYO_3_	3	1.20	2067	25.1	0.530	[147]
LuYO_3_	3	0.440	2074	-	0.140	[59]
YO_3_	3	0.603	2060	33.2	0.250	[52]
LuYO_3_	3	0.600	2076	11.5	0.250	[52]
(Sc_1/4_Y_3/4_)O_3_	5	1.24	2077	9.45	3.49	[53]
LuScO_3_ crystal	1	0.250	1982	55	0.038	[143]
LuScO_3_ crystal	1	0.705	2100	55	0.038	[143]

**Table 3:** Data obtained in the pulsed regime of the mixed ceramics reported in the literature; λ_L_: laser wavelength emission; P_out_: laser output power; τ_L_: pulse duration; ***f***: repetition rate.

Sample	λ_L_ (nm)	P_out_ (mW)	τ_L_ (fs)	*f* (MHz)	Ref.
LuScO_3_	1975	32	590 ps	34.72	[55]
Lu_2/3_Sc_1/3_O_3_	2057	30	63	78.9	[54]
Lu_2/3_Sc_1/3_O_3_	2081	220	58	84.8	[56]
LuYO_3_	2048	51	54	78	[52]
LuYO_3_	2045	63	57	72.6	[59]
LuYO_3_	2061	121	410	139.3	[147]
LuYO_3_	2047	540	120.3 ns	26.31	[51]
LuScO_3_^c^	2093	113	170	115.2	[146]

## Data Availability

Not applicable.
